# Food insecurity is associated with chronic pain and high-impact chronic pain in the USA

**DOI:** 10.1017/S1368980023002732

**Published:** 2023-12-13

**Authors:** Javier A Tamargo, Larissa J Strath, Shama D Karanth, Antoinette L Spector, Kimberly T Sibille, Stephen Anton, Yenisel Cruz-Almeida

**Affiliations:** 1 Pain Research and Intervention Center of Excellence, University of Florida, Gainesville, FL, USA; 2 Institute on Aging, University of Florida, Gainesville, FL, USA; 3 Department of Community Dentistry and Behavioral Science, University of Florida, Gainesville, FL, USA; 4 University of Florida Health Cancer Center, University of Florida, Gainesville, FL, USA; 5 College of Health Professions and Sciences, University of Wisconsin-Milwaukee, Milwaukee, WI, USA; 6 Department of Physical Medicine & Rehabilitation, College of Medicine, University of Florida, Gainesville, FL, USA

**Keywords:** Food insecurity, Social determinants of health, Chronic pain, High-impact chronic pain, Health disparities, Vulnerable populations

## Abstract

**Objective:**

This study evaluated whether food insecurity (US Adult Food Security Survey) was associated with chronic pain (≥ 3 months) and high-impact chronic pain (i.e. pain that limits work and life) among US adults.

**Design:**

Cross-sectional analysis.

**Setting:**

Nationally representative sample of non-institutionalised adults in the USA.

**Participants:**

79 686 adults from the National Health Interview Survey (2019–2021).

**Results:**

Marginal, low and very low food security were associated with increased prevalence odds of chronic pain (OR: 1·58 (95 % CI 1·44, 1·72), 2·28 (95 % CI 2·06, 2·52) and 3·37 (95 % CI 3·01, 3·78), respectively) and high-impact chronic pain (OR: 1·28 (95 % CI 1·14, 1·42), 1·55 (95 % CI 1·37, 1·75) and 1·90 (95 % CI 1·65, 2·18), respectively) in a dose–response fashion (*P*-trend < 0·0001 for both), adjusted for sociodemographic, socio-economic and clinically relevant factors. Participation in Supplemental Nutrition Assistance Program (SNAP) and age modified the association between food insecurity and chronic pain.

**Conclusions:**

These findings illustrate the impact of socio-economic factors on chronic pain and suggest that food insecurity may be a social determinant of chronic pain. Further research is needed to better understand the complex relationship between food insecurity and chronic pain and to identify targets for interventions. Moreover, the consideration of food insecurity in the clinical assessment of pain and pain-related conditions among socio-economically disadvantaged adults may be warranted.

Chronic pain, defined as pain that persists for more than 3 months^(1)^, is among the most common chronic conditions and leading causes of disability in the USA^(2)^. Over 20 % of US adults suffer from chronic pain and up to half of those (7–10 % of US adults) report high-impact chronic pain, defined as pain that frequently limits life and work activities^(3,4)^. Alarmingly, the proportion of US adults reporting pain and painful health conditions has been on the rise^(5–7)^. While chronic pain frequently co-occurs with an underlying disease, it is also recognised as an independent condition^(1,8)^.

Chronic pain is a highly complex and multidimensional condition, with dynamic interplays of biological, psychological, behavioural, sociocultural and environmental factors contributing to its development and progression^(9–11)^. Certain population subgroups are especially vulnerable to chronic pain, particularly older adults and those who experience socio-economic disadvantages^(10)^. Notably, much of the heterogeneity in the epidemiology of chronic pain can be explained by socio-economic factors, such as income and education^(3,5,7,12)^. As such, the influence of socio-economic factors on chronic pain, and vice versa, is an increasingly important area of investigation, and further research on mechanisms underlying socio-economic disparities in chronic pain is needed.

Food insecurity is recognised as a social determinant of health with overwhelming evidence linking it to an increased burden of chronic diseases and mental health disorders^(13–15)^. Food insecurity refers to limited or uncertain access to sufficient nutritious foods. It may be the result of financial constraints, limited availability of food choices (e.g. food deserts) or difficulties accessing food (e.g. lack of transportation). Food insecurity is not to be confused with hunger, a physiologic condition that may result from severe food insecurity. According to the US Department of Agriculture (USDA), 10·2 % of US households (13·5 million) experienced food insecurity during 2021^(16)^. Food insecurity affects some of the most vulnerable individuals in the USA and has the potential to influence many of the factors that contribute to chronic pain. Notably, the impact of food insecurity on health outcomes is often independent of, additive to, and/or greater than other socio-economic risk factors, such as low income^(13,15,17)^. On the other hand, food insecurity may be more modifiable than other socio-economic factors, for instance, through food assistance programmes.

Despite the growing recognition of food insecurity as a significant risk factor for many of the most prevalent public health concerns^(13)^, relatively little attention has been given to the potential link between food insecurity and chronic pain. A growing body of evidence has associated food insecurity with an increased risk of pain^(17)^ and pain-related emergency room visits^(18)^ in Canada. In the USA, one study observed that 53 % of food bank users reported chronic pain^(19)^. However, population-based studies of chronic pain and high-impact chronic pain in relation to food insecurity in the USA are lacking.

Given the immense public health burden of chronic pain and the potential link with food insecurity, this study evaluated the relationship between food insecurity and chronic pain among US adults using US population-based data. We also evaluated the relationship between food insecurity and high-impact chronic pain, a US National Pain Strategy and Healthy People 2030 priority due to its interference with and limiting impact on people’s lives.

## Methods

### Study design and population

**Fig. 1:**
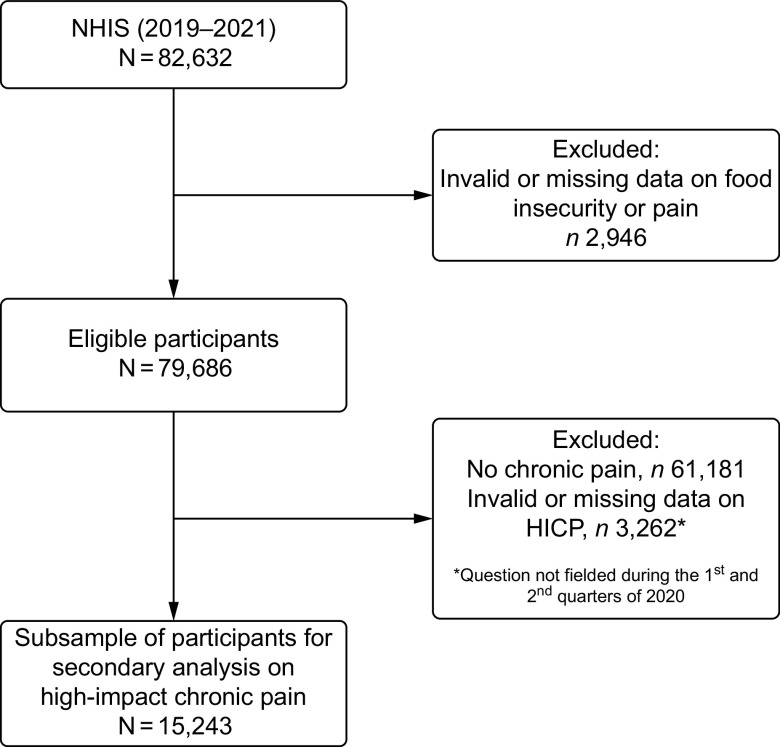
Participant flow chart

This cross-sectional study utilised pooled data from the 2019–2021 National Health Interview Survey (NHIS)^(20)^ to investigate the relationship between food insecurity and chronic pain. The NHIS is a nationally representative household survey of the US civilian non-institutionalised population residing within the fifty states and the District of Columbia. The NHIS is conducted annually by the National Center for Health Statistics (NCHS), which is part of the Centers for Disease Control and Prevention (CDC). A detailed description of the NHIS sampling methodology and data collection procedures is available on their website (https://www.cdc.gov/nchs/nhis/index.htm). Participants with missing or invalid data (refused, not ascertained, don’t know) on food security or pain were excluded from this study (*n* 2946). A flow chart for inclusion and exclusion of participants can be seen in Fig. 1.

### Food insecurity

The ten-item US Adult Food Security Survey was used to measure food security status in the past 30 d^(16)^. Based on the number of affirmative responses, participants were classified as having high (0), marginal (1–2), low (3–5) or very low food security (6–10). These categories correspond to the following constructs, as defined by the USDA:

- High food security: no indications of food-access problems or limitations.
- Marginal food security: some anxiety over food sufficiency, with little or no indication of changes in diets or food intake.
- Low food security: reduced quality, variety, or desirability of diet, with little or no indication of reduced food intake.
- Very low food security: multiple indications of disrupted eating patterns and reduced food intake.

Regarding the use of food assistance programmes, participants were asked whether they had received Supplemental Nutrition Assistance Program (SNAP) benefits, formerly known as ‘food stamps’, in the past 12 months (yes, no).

### Chronic pain and related outcomes

The primary outcome of this study was the presence of chronic pain. Participants were asked: ‘In the past three months, how often did you have pain? Would you say never, some days, most days, or every day?’ Chronic pain was defined as having pain ‘most days’ or ‘every day’ in the past 3 months^(3,4)^. The presence of high-impact chronic pain among the subset of participants who reported chronic pain was a secondary outcome of this study. Participants who reported pain at least ‘some days’ were also asked: ‘Over the past three months, how often did your pain limit your life or work activities? Would you say never, some days, most days, or every day?’ Participants classified as having chronic pain who also reported limiting pain ‘most days’ or ‘every day’ were classified as having high-impact chronic pain^(3)^. This question was not fielded during the first and second quarters of the 2020 NHIS, thus it is missing in a subset of participants (*n* 3251) who reported chronic pain during the 2020 NHIS but were not asked about high-impact pain.

### Additional explanatory variables

Both food insecurity and chronic pain are highly complex, multifactorial issues associated with several shared social, behavioural, psychological and biological factors. We therefore adjusted for potential confounding factors, including sociodemographic variables: age (18–44, 45–64 and ≥ 65 years), sex (male and female), race/ethnicity (Hispanic, non-Hispanic (NH) White, NH Black/African American, NH Asian and other), household size (1 adult, ≥ 2 adults; no children and ≥ 1 child), marital status (married, widowed, divorced or separated, never married, living with a partner), US veteran (yes and no); socio-economic: US citizenship (yes and no), US native (yes and no), educational attainment (less than high school, high school or equivalent, some college, bachelor’s degree or higher), household income (< 100 %, 100 to < 200 %, 200 to < 400 %, ≥ 400 % of federal poverty level (FPL)), employment (employed, not employed but worked previously and never worked), health insurance (private, Medicaid and other public coverage, other coverage, and uninsured), US geographical region (Northeast, Midwest, South and West), urbanisation (large central metro, large fringe metro, medium and small metro, and non-metropolitan); and clinically relevant factors: smoking (never, current smoker and former smoker), BMI (BMI, underweight (< 18·5 kg/m^2^), healthy weight (18·5–24·9 kg/m^2^), overweight (25·0–29·9 kg/m^2^) and obesity (≥ 30·0 kg/m^2^)), and diagnosis (yes, no) of: arthritis, asthma, cancer, CHD, chronic obstructive pulmonary disease/emphysema/chronic bronchitis, hypertension, diabetes, myocardial infarction, stroke, dementia, anxiety and depression. Previous studies using NHIS data have reported on the relationship between these factors and the prevalence of chronic pain and/or high-impact chronic pain^(3,12,21,22)^.

### Statistical analysis

Sample characteristics are presented by food security status as number of participants and weighted percentages. Differences between levels of food security (high, marginal, low and very low) were tested with Rao–Scott chi-square test. Weighted logistic regressions for chronic pain and high-impact chronic pain were performed, adjusting for the complex survey design (i.e. stratified cluster sampling), sampling weights provided by the NHIS, and year of survey to estimate adjusted OR and 95 % CI. Multivariable logistic regression models were also performed in order to adjust for additional explanatory variables, including sociodemographic, socio-economic and clinically relevant factors, as well as SNAP participation. Trend tests for food insecurity were conducted by treating food insecurity as an ordinal variable in the multivariable logistic regression model. We also explored potential interaction effects between food insecurity and significant covariates. Results were considered statistically significant at *P* < 0·05. The data analysis for this paper was generated using SAS software, version 9.4 of the SAS System for Windows. Copyright © (2013) SAS Institute Inc. SAS and all other SAS Institute Inc. product or service names are registered trademarks or trademarks of SAS Institute Inc., Cary, NC, USA.

## Results

### Sample characteristics

Sample characteristics can be found in Table 1. The analytic sample consisted of 79 686 US adults of ages 18–44 (45·8 %), 45–64 (32·5 %) and ≥ 65 (21·6 %) years. Overall, participants were 51·6 % female, 63·4 % NH White, 11·5 % NH Black and 16·6 % Hispanic. Most participants lived in households of ≥ 2 adults (81·5 %), were a US citizen (91·7 %), and US native (81·6 %) and lived in urban areas (86·1 %). Ten per cent of participants fell under the FPL for household income, while 41·5 % had incomes of 400 % or higher. Over a third of participants had a high school level education or lower (38·5 %). Most participants were overweight (33·2 %) or had obesity (31·9 %) with a wide range of chronic health conditions ranging from history of stroke (2·8 %) to hypertension (31·2 %). Additionally, 16·6 % and 15·2 % reported a diagnosis of depression and anxiety, respectively.

**Table 1:** National Health Interview Survey (NHIS) 2019–2021 participants’ characteristics by food security level*

	Overall		High food security		Marginal food security		Low food security		Very low food security		*P*-value
	*n*	%	*n*	%	*n*	%	*n*	%	*n*	%	
*n* (weighted %)	79 686	100	70 151	86·8	4029	5·8	3171	4·4	2335	3·1	–
Age (years)											<0·0001
18–44	29 477	45·8	25 256	44·7	1813	54·1	1356	49·1	1052	54·9	
45–64	26 339	32·5	22 850	32·3	1357	31·2	1200	36·4	932	34·3	
≥65	23 698	21·6	21 886	22·8	852	14·5	612	14·3	348	10·7	
Sex											<0·0001
Female	43 151	51·6	37 322	50·7	2435	56·7	1992	59·9	1402	57·7	
Race/ethnicity											<0·0001
Hispanic	10 398	16·6	8347	15·1	914	26·8	764	28·7	373	20·7	
NH White	54 511	63·4	49 801	66·4	2005	44·3	1439	39·6	1266	49·2	
NH Black	8358	11·5	6389	10·0	756	19·1	697	22·8	516	22·5	
NH Asian	4422	5·9	4072	6·2	188	5·6	113	3·8	49	2·3	
Other	1997	2·6	1542	2·3	166	4·2	158	5·1	131	5·3	
Household											
≥2 adults	51 625	81·5	46 357	82·3	2412	79·7	1759	76·2	1097	70·3	<0·0001
≥1 child	21 341	33·2	18 021	31·8	1491	44·0	1122	42·3	707	38·6	<0·0001
Household income†											<0·0001
<100 % FPL	7913	10·1	4817	7·0	1063	24·3	1088	32·4	945	39·5	
100–200 % FPL	13 873	18·1	10 444	15·3	1381	34·5	1196	38·7	852	36·7	
200–400 % FPL	23 500	30·3	21 201	31·0	1164	31·4	688	22·7	447	19·8	
≥400 % FPL	34 400	41·5	33 689	46·7	421	9·8	199	6·3	91	3·9	
Marital status											<0·0001
Married	37 547	52·0	34 727	54·4	1361	39·5	952	36·9	507	27·8	
Widowed	7929	5·9	7103	5·9	375	6·2	262	6·0	189	5·4	
Divorced or separated	12 218	10·1	9993	9·2	781	13·0	755	15·9	689	20·3	
Never married	16 522	23·2	13 778	22·3	1125	29·2	900	28·3	719	32·6	
Living with a partner	5207	8·5	4318	7·9	376	11·8	291	12·7	222	13·6	
US-born	66 986	81·6	59 380	82·5	3135	74·2	2450	73·1	2021	82·1	<0·0001
US citizen	74 565	91·7	66 020	92·5	3584	86·3	2787	83·8	2174	88·7	<0·0001
Veteran status	7659	8·0	7073	8·4	252	4·9	183	4·8	151	5·1	<0·0001
Education											<0·0001
Less than high school	6810	11·0	4920	9·1	714	20·4	716	26·8	460	25·0	
High school or GED	19 779	27·5	16 640	26·4	1360	36·7	1048	33·4	731	33·1	
Some college	12 542	16·8	10 798	16·7	699	16·7	544	17·3	501	19·8	
Bachelor’s degree or higher	40 173	44·1	37 483	47·3	1232	25·3	833	21·3	625	21·3	
Employment											<0·0001
Employed	46 745	62·5	42 152	64·1	2129	57·1	1488	50·2	976	45·4	
Not employed, worked previously	31 304	34·6	26 688	33·2	1773	39·0	1562	44·7	1281	49·8	
Not employed, never worked	1501	2·7	1206	2·5	113	3·5	109	4·7	73	4·7	
Health insurance											<0·0001
Private	49 046	62·0	45 813	66·2	1605	40·5	1002	31·9	626	25·9	
Medicare or other public coverage	18 927	21·6	15 030	19·1	1501	34·4	1359	39·4	1037	42·2	
Other coverage	4924	5·3	4201	5·2	268	5·2	242	6·7	213	7·5	
Uninsured	6572	10·8	4923	9·2	641	19·4	555	21·7	453	24·1	
US region											<0·0001
Northeast	13 450	17·5	12 007	17·8	647	16·3	473	15·0	323	14·6	
Midwest	17 689	21·1	15 734	21·4	794	18·6	615	17·7	546	22·2	
South	28 617	37·7	24 632	36·8	1635	42·7	1358	45·1	992	44·1	
West	19 930	23·7	17 778	24·0	953	22·4	725	22·2	474	19·1	
Urban–rural											<0·0001
Large central metro	23 474	30·7	20 458	30·2	1282	33·4	1055	36·1	679	30·7	
Large fringe metro	18 737	24·6	16 941	25·3	826	22·0	563	17·6	407	19·1	
Medium and small metro	25 442	30·8	22 333	30·8	1306	30·0	1006	29·7	797	31·9	
Non-metropolitan	12 033	14·0	10 419	13·6	615	14·6	547	16·6	452	18·2	
Smoking history											<0·0001
Never	48 963	64·4	44 069	65·8	2266	59·0	1636	55·7	992	47·6	
Former	20 578	22·9	18 513	23·4	892	19·9	690	19·7	483	18·7	
Current	10 023	12·5	7469	10·7	863	20·9	838	24·5	853	33·4	
BMI											<0·0001
Underweight	1245	1·6	1079	1·6	76	1·9	39	1·2	51	2·8	
Healthy weight	24 828	30·9	22 451	31·9	1042	25·0	749	23·6	586	25·2	
Overweight	26 823	33·2	24 040	33·7	1222	31·9	939	29·5	622	26·0	
Obese	24 912	31·9	20 955	30·5	1581	38·8	1363	43·4	1013	42·8	
Medical conditions											
Anxiety	12 416	15·2	9564	13·5	957	21·3	930	26·3	965	36·2	<0·0001
Arthritis	20 506	21·1	17 355	20·3	1180	22·9	1081	27·6	890	31·9	<0·0001
Asthma	10 838	13·8	8830	12·9	719	17·2	676	20·0	613	24·7	<0·0001
Cancer	9850	9·7	8919	9·9	363	7·2	311	7·9	257	9·4	0·0002
COPD/emphysema/chronic bronchitis	4495	4·7	3435	4·0	354	6·7	334	8·4	372	13·9	<0·0001
CHD	4713	4·7	4019	4·5	245	4·9	243	6·4	206	7·4	<0·0001
Dementia	910	1·0	765	0·9	55	1·1	52	1·5	38	1·3	0·040
Depression	14 006	16·6	10 779	14·7	1075	23·4	1057	29·5	1095	41·8	<0·0001
Diabetes	8367	9·3	6830	8·6	567	12·2	555	15·2	415	15·6	<0·0001
High cholesterol	23 847	25·9	20 884	25·7	1182	25·2	1032	28·9	749	28·1	<0·0001
Hypertension	28 541	31·2	24 637	30·5	1520	32·5	1359	38·0	1025	38·5	<0·0001
Myocardial infarction	2950	3·0	2428	2·8	166	3·1	187	4·7	169	5·5	<0·0001
Stroke	2775	2·8	2205	2·5	196	3·7	204	5·4	170	6·0	<0·0001
Food assistance											
SNAP (past 12 months)	8445	11·8	4830	7·9	1284	31·5	1289	41·3	1042	44·3	<0·0001

NH, non-Hispanic; FPL, federal poverty level; GED, General Educational Development; COPD, chronic obstructive pulmonary disease; SNAP, Supplemental Nutrition Assistance Program.

* Percentages shown are adjusted for complex survey design and NHIS sampling weights. Column percentages may not aggregate to 100 per cent.

† Annual household income is reported as a percentage of the FPL.

### Food insecurity

In total, 13·3 % of participants reported some level of food insecurity, with 5·8 %, 4·4 % and 3·1 % reporting marginal, low and very low food security, respectively. Additionally, 11·8 % of participants reported receiving SNAP benefits with a higher proportion among food-insecure participants, ranging from 31·5 % to 44·3 % between those with marginal and very low food security, compared with 7·9 % among those with high food security.

### Food insecurity and chronic pain

**Fig. 2:**
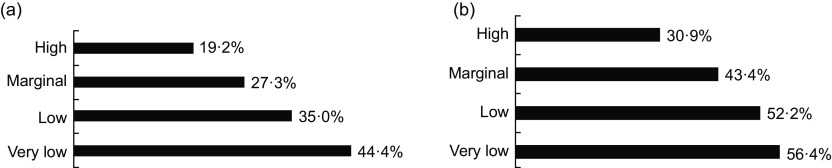
(a) Chronic pain by food security level (*n* 79 686). (b) High-impact chronic pain by food security level in adults with chronic pain (*n* 15 243)

A total of 21·1 % of participants reported chronic pain. The prevalence of chronic pain was incrementally higher as the severity of food insecurity increased (Fig. 2). Compared with 19·2 % among adults with high food security, the rates of chronic pain for marginal, low and very low food security were 27·3 %, 35·0 % and 44·4 %, respectively. Indeed, food insecurity was associated with increased risk of chronic pain at all levels and a significant dose–response effect (*P* < 0·0001 for trend); see Table 2. Compared with high food security, the odds of chronic pain were 1·58 (95 % CI 1·44, 1·72), 2·27 (95 % CI 2·06, 2·52) and 3·37 (95 % CI 3·01, 3·78) for those with marginal, low and very low food security, respectively. These relationships remained consistent, although the effect size was attenuated, after adjustment for covariates: 1·28 (95 % CI 1·14, 1·42), 1·55 (95 % CI 1·37, 1·75) and 1·90 (95 % CI 1·65, 2·18) for marginal, low and very low food security, respectively.

**Table 2:** Associations between food insecurity and chronic pain

	*n*	Weighted %	Model 1*		Model 2†	
			aOR	95 % CI	aOR	95 % CI
Chronic pain (*n* 79 686)						
High food security	14 808	19·2	Reference		Reference	
Marginal food security	1254	27·3	**1·58**	**1·44, 1·72**	**1·28**	**1·14, 1·42**
Low food security	1265	35·0	**2·28**	**2·06, 2·52**	**1·55**	**1·37, 1·75**
Very low food security	1178	44·4	**3·37**	**3·01, 3·78**	**1·90**	**1·65, 2·18**
			*P*-trend < 0·0001		*P*-trend < 0·0001	
High-impact chronic pain (*n* 15 243)						
High food security	3862	30·9	Reference		Reference	
Marginal food security	447	43·4	**1·71**	**1·44, 2·03**	**1·24**	**1·01, 1·52**
Low food security	555	52·2	**2·42**	**2·04, 2·88**	**1·47**	**1·21, 1·79**
Very low food security	584	65·4	**2·89**	**2·41, 3·43**	**1·70**	**1·38, 2·09**
			*P*-trend < 0·0001		*P*-trend < 0·0001	

aOR, adjusted OR; NHIS, National Health Interview Survey.

Bolded values denote statistical significance at *P* < 0·05.

* Model 1 is adjusted for complex survey design, NHIS sampling weights and survey year.

† Model 2 is additionally adjusted for age, sex, race/ethnicity, household size, household income, marital status, US-born, US citizenship, US veteran status, education, employment, health insurance, urban/rural residence, US region, smoking, BMI, arthritis, asthma, cancer, CHD, chronic obstructive pulmonary disease/emphysema/chronic bronchitis, high cholesterol, hypertension, diabetes, myocardial infarction, stroke, dementia, anxiety, depression and SNAP participation (also known as ‘food stamps’).

Food insecurity, particularly at the levels of low and very low food security, showed a stronger association with chronic pain than other socio-economic factors, such as household income (aOR: 1·32 (95 % CI 1·17, 1·48) for income < 100 % FPL compared with ≥ 400 % FPL), education (aOR: 1·11 (95 % CI 1·00, 1·24) for ‘less than high school’ compared with ‘bachelor’s degree or higher’), employment (aOR: 1·26 (95 % CI 1·18, 1·35) for ‘not employed, worked previously’ compared with employed) and rural residence (aOR: 1·07 (95 % CI 0·98, 1·17) compared with large central metro). The full model with estimates for all the independent variables can be found in online supplementary material, Supplemental Table 1.

### The relationship between food insecurity and chronic pain is modified by SNAP and age

We also explored potential interaction effects between food insecurity and covariates that were found to be significantly associated with chronic pain. There was a significant interaction effect between food insecurity and SNAP participation on chronic pain (*P* = 0·015). To better illustrate this effect, in Table 3, we show the association of food insecurity and chronic pain stratified by SNAP participation. Among SNAP non-participants, all levels of food insecurity were associated with chronic pain. However, among SNAP participants, only very low food security was associated with chronic pain, but not marginal and low food security levels. Additionally, we found a significant interaction effect between food insecurity and age (*P* < 0·0001), as shown in Table 4. Whereas all levels of food insecurity were associated with chronic pain among 18–64-year-olds, only low and very low food security (not marginal food security) remained significantly associated with chronic pain in older adults.

**Table 3:** SNAP use modifies the association of food insecurity with chronic pain and high-impact chronic pain

Outcome	Food security level	SNAP non-participants				SNAP participants				*P*-interaction*
		*n*	Weighted %	aOR†	95 % CI	*n*	Weighted %	aOR	95 % CI	
Chronic pain (*n* 79 686)										
	High food security	65 033	90·7	Reference		4830	58·0	Reference		0·015
	Marginal food security	2271	4·4	**1·37**	**1·19, 1·57**	1284	15·3	1·08	0·88, 1·33	
	Low food security	1863	2·9	**1·83**	**1·58, 2·12**	1289	15·2	1·19	0·97, 1·45	
	Very low food security	1288	1·9	**1·87**	**1·56, 2·24**	1042	11·4	**1·84**	**1·48, 2·28**	
High-impact chronic pain (*n* 15 243)										
	High food security	10 869	85·9	Reference		1310	50·3	Reference		<0·0001
	Marginal food security	604	5·5	**1·38**	**1·08, 1·75**	388	14·5	1·03	0·74, 1·43	
	Low food security	569	4·9	**1·35**	**1·08, 1·70**	471	17·1	**1·56**	**1·12, 2·17**	
	Very low food security	472	3·6	**1·84**	**1·41, 2·40**	494	18·0	**1·54**	**1·10, 2·15**	

SNAP, Supplemental Nutrition Assistance Program; NHIS, National Health Interview Survey.

Bolded values denote statistical significance at *P* < 0·05.

* *P*-value for food insecurity*SNAP participation interaction term.

† OR and 95 % CI are adjusted for age, sex, race/ethnicity, household size, household income, marital status, US-born, US citizenship, US veteran status, education, employment, health insurance, urban/rural residence, US region, smoking, BMI, arthritis, asthma, cancer, CHD, chronic obstructive pulmonary disease/emphysema/chronic bronchitis, high cholesterol, hypertension, diabetes, myocardial infarction, stroke, dementia, anxiety, depression, as well as complex sampling design, NHIS sampling weights and survey year.

**Table 4:** Age modifies the association of food insecurity with chronic pain and high-impact chronic pain

Outcome	Food security level	18–44 years*		45–64 years*		≥ 65 years*		*P*-interaction†
		OR	95 % CI	OR	95 % CI	OR	95 % CI	
Chronic pain (*n*, weighted %)		3784	12·4	7213	26·7	7488	31·2	
(*n* 79 686)	High food security	Reference		Reference		Reference		<0·0001
	Marginal food security	**1·33**	**1·12, 1·59**	**1·27**	**1·08, 1·50**	1·16	0·92, 1·47	
	Low food security	**1·35**	**1·12, 1·64**	**1·75**	**1·44, 2·12**	**1·67**	**1·29, 2·16**	
	Very low food security	**1·74**	**1·43, 2·13**	**2·08**	**1·66, 2·60**	**2·00**	**1·42, 2·83**	
High-impact chronic pain (*n*, weighted %)		845	26·3	2365	39·4	2232	36·5	
(*n* 15 243)	High food security	Reference		Reference		Reference		<0·0001
	Marginal food security	1·31	0·90, 1·91	1·23	0·92, 1·63	1·15	0·80, 1·64	
	Low food security	1·29	0·89, 1·86	**1·67**	**1·31, 2·17**	1·28	0·90, 1·82	
	Very low food security	1·42	0·98, 2·05	**1·84**	**1·37, 2·46**	**2·13**	**1·34, 3·37**	

SNAP, Supplemental Nutrition Assistance Program; NHIS, National Health Interview Survey.

Bolded values denote statistical significance at *P* < 0·05.

* OR and 95 % CI are adjusted for sex, race/ethnicity, household size, household income, marital status, US-born, US citizenship, US veteran status, education, employment, health insurance, urban/rural residence, US region, smoking, BMI, arthritis, asthma, cancer, CHD, chronic obstructive pulmonary disease/emphysema/chronic bronchitis, high cholesterol, hypertension, diabetes, myocardial infarction, stroke, dementia, anxiety, depression, SNAP participation, as well as complex sampling design, NHIS sampling weights and survey year.

† *P*-value for food insecurity*age interaction term.

### Food insecurity and high-impact chronic pain

Over a third (35·0 %) of participants who reported chronic pain (and were asked about high-impact pain) also reported high-impact chronic pain. As seen previously, there was a direct correlation between the severity of food insecurity and high-impact chronic pain prevalence (Fig. 2, Table 2). Compared with 30·9 % among adults with high food security, the rates of high-impact chronic pain for marginal, low and very low food security were 43·4 %, 52·2 % and 56·4 %, respectively. Compared with high food security, the odds of high-impact chronic pain in relation to marginal, low and very low food security were 1·71 (95 % CI 1·44, 2·03), 2·44, (95 % CI 2·04, 2·88) and 2·89 (95 % CI 2·41, 3·43), respectively; *P*-trend < 0·0001. After adjustment for covariates, these effect sizes were attenuated but remained significantly associated with high-impact chronic pain (aOR: 1·24 (95 % CI 1·01, 1·52), 1·47 (95 % CI 1·21, 1·79) and 1·70 (95 % CI 1·38, 2·09) for marginal, low and very low food security, respectively, as compared with high food security). Similar to chronic pain, food insecurity showed a stronger association with high-impact chronic pain than other socio-economic factors, except for employment (aOR: 2·16 (95 % CI 1·47, 3·17) for ‘not employed, never worked’ and 2·47 (95 % CI 2·19, 2·79) for ‘not employed, worked previously’ as compared with employed); see online supplementary material, Supplemental Table 1.

### The relationship between food insecurity and high-impact chronic pain is modified by SNAP and age

In the effect modification analyses, we found a significant interaction effects between food insecurity and SNAP participation (*P* < 0·0001) and age (*P* < 0·0001) on high-impact chronic pain. Among SNAP non-recipients, all levels of food insecurity were associated with high-impact chronic pain (Table 3). On the other hand, among SNAP recipients, only low and very low food security, but not marginal food security, remained significantly associated with high-impact chronic pain. With regard to age (Table 4), food insecurity was not associated with high-impact chronic pain among 18–44-year-olds. Among individuals aged 45–64 years, low and very low food security, but not marginal food security, were associated with high-impact chronic pain. Among participants ≥ 65 years old, only very low food security was associated with high-impact chronic pain.

## Discussion

This study evaluated the relationship between food insecurity and chronic pain in a large representative sample of US adults using pooled data from the 2019–2021 NHIS. The results suggest that food insecurity is a significant risk factor for chronic pain and high-impact chronic pain, independent of and with a stronger association than other socio-economic risk factors, such as income and education. Moreover, the odds of chronic pain and high-impact chronic pain increased in accordance with the severity of food insecurity, with individuals experiencing very low food security having the highest risk compared with those with high food security. These findings illustrate the impact of food insecurity as an important socio-economic factor that may influence chronic pain. The results regarding high-impact chronic pain, defined by frequent limitations to an individual’s life and work, are of particular importance and public health relevance. Indeed, reducing the prevalence of high-impact chronic pain is an objective within the US National Pain Strategy and Healthy People 2030. Interestingly, our findings also suggest that food assistance programmes (i.e. SNAP) may have a beneficial impact on chronic pain and high-impact chronic pain among individuals with marginal levels of food insecurity. Further research on socio-economic determinants of chronic pain is warranted, as well as the consideration of food insecurity in the clinical assessment of pain and pain-related conditions among socio-economically disadvantaged adults.

As shown in this study and others, over 20 % of US adults have chronic pain^(3)^. Our findings show a disproportionately high prevalence of chronic pain among the food-insecure population, with over a third (33·8 %) of food-insecure adults (those with at least marginal food security) reporting chronic pain. In comparison, the age-adjusted prevalence of chronic pain among individuals living under the FPL is 28·8 %^(3)^. Our findings add to previous reports by Men et al. showing that food insecurity was associated with pain^(17)^ and pain-related emergency room visits^(18)^ in the Canadian population. Similar to the present study, the investigators found a dose–response association between the severity of food insecurity and the odds of chronic pain and pain that prevents most activity in a nationally representative sample of the Canadian population^(17)^. Moreover, in both studies, the odds of pain outcomes in relation to food insecurity were markedly higher than other socio-economic factors, such as income and education. Our results may be more conservative than those by Men et al., as their study defined chronic pain as being ‘usually free of pain or discomfort’ rather than the temporal criterion of 3 months or longer as developed by the International Association for the Study of Pain (IASP)^(1)^. Additionally, the Canadian Community Health Survey uses a modified version of the US Household Food Security Survey and differences in the implementation and classification of food insecurity lead to underestimation of food insecurity in the USA as compared with Canada^(23)^.

Given that food insecurity affects over 10 % of US households^(16)^, the intersection of food insecurity and chronic pain represents a significant public health challenge. Of particular concern is the impact of food insecurity on older adults, a rapidly growing population group that is disproportionately affected by chronic pain^(3)^ and is vulnerable to food insecurity and compromised nutritional status^(24–26)^. Interestingly, we observed a modifying effect of age on the relationship between food insecurity and chronic pain. Among adults aged 18–44 years, food insecurity was associated with higher odds of chronic pain, but not high-impact chronic pain. This may be partly due to high-impact chronic pain being most predominant among adults 45 years of age and older^(3,21)^.

Yet, in older adults, only low and very low food security were associated with chronic pain or high-impact chronic pain, suggesting that older adults may be less susceptible to the impact of marginal levels of food insecurity on their burden of chronic pain. Nevertheless, the strength of the association between low/very low food security and pain outcomes was higher for older adults than for younger adults. The interaction between food insecurity and age may reflect that older adults in the USA are particularly vulnerable to severe food insecurity when living alone^(16)^, and that the prevalence of chronic pain, especially high-impact chronic pain, rises dramatically with age^(3,21)^. It may also reflect currently unknown factors including interactions with other socio-economic factors not measured in our study. Further research is needed to comprehend the complex interplay between food insecurity, age and chronic pain.

There are several potential mechanisms by which food insecurity may influence chronic pain, all of which relate to the complex interplay between shared social, behavioural and biological factors. Among these, food insecurity contributes to poor quality diets and maladaptive eating patterns that compromise nutritional status^(27–29)^. Thereby, food insecurity potentially exacerbates proposed mechanisms underlying the impact of nutrition on chronic pain, including malnutrition, obesity, inflammation, metabolic dysfunction and nervous system sensitisation, among others^(30,31)^. Notably, food insecurity has been associated with markers of systemic inflammation^(32)^, as well as diets with higher inflammatory potential^(33)^, which in turn have been associated with incidence of pain in middle-aged and older adults^(34,35)^.

The current study’s findings also suggest that SNAP participation may beneficially modify the association of food insecurity with chronic pain. Formerly known as the Food Stamp Program, SNAP is the largest food assistance programme in the USA. Yet, there has been limited research examining its impact on health outcomes. Promising findings include better self-reported health^(36)^, and reduced hospital admissions, healthcare costs, and mortality^(37,38)^. On the other hand, SNAP participants demonstrate poorer quality diets and higher likelihood of metabolic syndrome when compared with non-participant peers^(39,40)^. Although most eligible individuals participate in the programme (78 % in 2020)^(41)^, many SNAP recipients remain food-insecure even after receiving benefits^(42,43)^. Furthermore, the current criteria for eligibility, such as having a gross income below 130 % of the poverty line, prevent many individuals from obtaining SNAP benefits^(42)^. More research is needed to evaluate how food assistance programmes may play a role in lessening the burden of food insecurity, including its impact on chronic pain.

It is important to note that the relationship between food insecurity and chronic pain is likely bidirectional. Pain (and analgesic medications) can lead to loss of appetite and other gastrointestinal complications that compromise dietary intake and nutritional status. Similarly, chronic pain can lead to significant physical and functional limitations affecting the ability to secure food, particularly among older adults^(44)^. Inability to work and pain-related healthcare costs can result in financial hardships that contribute to food insecurity. Indeed, data from the 2011 NHIS showed that 83 % of participants with high-impact chronic pain were unable to work outside of the home^(21)^. Moreover, an analysis of the 2008 Medical Expenditure Panel Survey showed that chronic pain places a significant burden on the US economy, comprised of healthcare costs and reduced worker productivity, totalling between $560 and $635 billion annually^(45)^. This relationship may create a vicious cycle between chronic pain and food insecurity that leads to the deterioration of health and quality of life. Therefore, there is a need for improved interventions to secure access to sufficient nutritious foods among socio-economically disadvantaged groups, especially those who are subject to disability due to their pain.

### Strengths and limitations

The use of 2019–2021 NHIS data and its chronic pain supplement is a strength of this study, as the NHIS is the primary source of pain surveillance in the USA and the questions have been specifically designed based on IASP criteria for chronic pain and high-impact chronic pain. Additionally, the US Household Food Security Survey – used by the USDA to monitor food insecurity in the US population annually – is the most widely used and validated assessment of food insecurity^(46)^. On the other hand, this instrument does not entirely capture the experience of food insecurity, trading comprehensiveness for simplicity^(47)^. There may be factors unaccounted for in this analysis that may help explain the relationships seen in this study, such as dietary quality and structural barriers to food access or health care, among others. The COVID-19 pandemic may have influenced the results of the study. For one, the NHIS transitioned from primarily in-person to telephone interviews during 2020 and part of 2021. While NHIS weighing procedures minimise coverage and non-response bias, some measurement biases remain. Additionally, the early stages of the COVID-19 pandemic saw a temporary rise in food insecurity within the USA^(48)^, although, overall, the prevalence of food insecurity in the USA remained stable during 2019–2021^(16)^. While the cross-sectional study design does not allow for causality to be established, the dose–response effect between the severity of food insecurity and the odds of chronic pain suggests that food insecurity may at least partially contribute to chronic pain. Nevertheless, a bidirectional relationship is possible. For instance, longer episodes of chronic pain may lead to more severe disability and financial strains that contribute to more severe food insecurity.

### Conclusion

Food insecurity may be a social determinant of chronic pain among US adults. Food assistance programmes may provide a beneficial impact with regard to chronic pain among people with marginal levels of food insecurity. Further studies are needed to better understand the complex and temporal relationship between food insecurity and chronic pain and to identify targets for interventions. The findings of this study illustrate the impact of food insecurity on chronic pain based on cross-sectional analyses. Studies are needed using longitudinal designs to further explore how food insecurity may influence chronic pain over time. Furthermore, the consideration of socio-economic factors such as food insecurity in the clinical assessment of pain and pain-related conditions among socio-economically disadvantaged adults may be warranted.

## Supporting information

Tamargo et al. supplementary material Tamargo et al. supplementary material

## Supplementary material

For supplementary material accompanying this paper visit https://doi.org/10.1017/S1368980023002732
