# Supplementary material for: Food insecurity is associated with chronic pain and high-impact chronic pain in the USA
Source: Public Health Nutr. 2023 Dec 13;27(1):e7. doi: 10.1017/S1368980023002732 (PMC10830368; doi:10.1017/S1368980023002732)
Supplement: Tamargo et al. supplementary material [file S1368980023002732sup001.docx]

**Supplementary Table 1. Multivariable logistic regressions for chronic pain and high-impact chronic pain among NHIS 2019-2021 participants.**

| Variable | Category | Reference | Chronic pain  (N=79,686) |  | High-impact chronic pain  (N=15,243) |
| --- | --- | --- | --- | --- | --- |
|  |  |  | **aOR (95% CI)^1^** |  | **aOR (95% CI)^1^** |
| Age (years) | 45-64 | 18-44 | 1.51 (1.40, 1.63) |  | 1.41 (1.21, 1.64) |
|  | 65+ |  | 1.06 (0.96, 1.17) |  | 0.85 (0.70, 1.03) |
| Sex | Female | Male | 0.98 (0.92, 1.03) |  | 1.04 (0.93, 1.16) |
| Race/ethnicity | Hispanic | NH White | 0.76 (0.69, 0.84) |  | 1.09 (0.91, 1.31) |
|  | Black/African American |  | 0.76 (0.70, 0.83) |  | 1.05 (0.88, 1.25) |
|  | Asian |  | 0.55 (0.46, 0.65) |  | 1.05 (0.66, 1.69) |
|  | Other |  | 1.07 (0.88, 1.30) |  | 1.12 (0.85, 1.47) |
| Household size | 2+ adults | 1 adult | 1.02 (0.95, 1.10) |  | 1.06 (0.93, 1.22) |
|  | 1+ children | No children | 1.06 (0.99, 1.14) |  | 1.02 (0.89, 1.18) |
| Household income | <100% FPL | >400% FPL | 1.32 (1.17, 1.48) |  | 1.20 (0.99, 1.46) |
|  | 100-200% FPL |  | 1.21 (1.11, 1.31) |  | 1.21 (1.04, 1.41) |
|  | 200-400% FPL |  | 1.18 (1.10, 1.26) |  | 1.20 (1.07, 1.35) |
| Marital status | Divorced or separated | Married | 1.06 (0.97, 1.16) |  | 1.20 (1.02, 1.41) |
|  | Living with a partner |  | 1.13 (1.02, 1.25) |  | 1.16 (0.96, 1.40) |
|  | Never married |  | 0.83 (0.76, 0.92) |  | 1.09 (0.91, 1.31) |
|  | Widowed |  | 0.91 (0.82, 1.01) |  | 1.15 (0.96, 1.38) |
| U.S. native | Yes | No | 1.25 (1.13, 1.38) |  | 0.87 (0.69, 1.10) |
| U.S. citizen | Yes | No | 1.12 (0.96, 1.31) |  | 0.83 (0.59, 1.16) |
| U.S. veteran | Yes | No | 1.11 (1.02, 1.22) |  | 1.06 (0.90, 1.25) |
| Education | Less than high-school |  | 1.11 (1.00, 1.24) |  | 1.07 (0.91, 1.26) |
|  | High-school or GED | Bachelor's degree or higher | 1.05 (0.98, 1.12) |  | 1.13 (1.00, 1.28) |
|  | Some college |  | 1.09 (1.01, 1.17) |  | 1.13 (0.99, 1.29) |
| Employment | Not employed, worked previously | Currently employed | 1.26 (1.18, 1.35) |  | 2.47 (2.19, 2.79) |
|  | Not employed, never worked |  | 0.86 (0.70, 1.05) |  | 2.16 (1.47, 3.17) |
| Health insurance | Medicaid and other public | Private | 1.06 (0.99, 1.14) |  | 1.04 (0.92, 1.19) |
|  | Other coverage |  | 1.40 (1.26, 1.54) |  | 1.30 (1.11, 1.53) |
|  | Uninsured |  | 1.01 (0.90, 1.12) |  | 0.97 (0.78, 1.21) |
| U.S. Region | Midwest | Northeast | 1.16 (1.05, 1.28) |  | 0.94 (0.79, 1.11) |
|  | South |  | 1.10 (1.00, 1.20) |  | 1.04 (0.88, 1.23) |
|  | West |  | 1.31 (1.19, 1.44) |  | 1.18 (0.98, 1.42) |
| Urban-Rural | Large fringe metro | Large central metro | 1.00 (0.93, 1.08) |  | 0.99 (0.85, 1.15) |
|  | Medium and small metro |  | 1.07 (1.00, 1.16) |  | 0.94 (0.82, 1.07) |
|  | Nonmetropolitan |  | 1.07 (0.98, 1.17) |  | 0.97 (0.82, 1.15) |
| Smoking | Current smoker | Never smoker | 1.65 (1.53, 1.78) |  | 1.09 (0.95, 1.25) |
|  | Former smoker |  | 1.32 (1.25, 1.40) |  | 1.03 (0.92, 1.16) |
| Body mass index | Underweight | Healthy weight | 1.13 (0.93, 1.37) |  | 1.52 (1.07, 2.15) |
|  | Overweight |  | 1.09 (1.02, 1.16) |  | 1.06 (0.93, 1.20) |
|  | Obese |  | 1.36 (1.28, 1.45) |  | 1.15 (1.02, 1.30) |
| Medical condition | Anxiety |  | 1.43 (1.33, 1.54) |  | 1.11 (0.98, 1.26) |
|  | Arthritis |  | 4.39 (4.16, 4.63) |  | 1.38 (1.23, 1.54) |
|  | Asthma |  | 1.36 (1.26, 1.46) |  | 1.23 (1.08, 1.40) |
|  | Cancer |  | 1.18 (1.10, 1.26) |  | 1.10 (0.97, 1.23) |
|  | COPD/emphysema/chronic bronchitis |  | 1.51 (1.37, 1.67) |  | 1.32 (1.14, 1.53) |
|  | Coronary heart disease |  | 1.19 (1.06, 1.33) |  | 1.07 (0.90, 1.27) |
|  | Dementia |  | 1.22 (0.99, 1.51) |  | 2.04 (1.50, 2.76) |
|  | Depression |  | 1.74 (1.62, 1.87) |  | 1.48 (1.30, 1.68) |
|  | Diabetes |  | 1.26 (1.17, 1.37) |  | 1.16 (1.02, 1.32) |
|  | High cholesterol |  | 1.14 (1.08, 1.21) |  | 0.93 (0.84, 1.03) |
|  | Hypertension |  | 1.21 (1.14, 1.28) |  | 1.18 (1.07, 1.31) |
|  | Myocardial infarction |  | 1.04 (0.91, 1.19) |  | 1.13 (0.93, 1.37) |
|  | Stroke |  | 1.22 (1.07, 1.38) |  | 1.25 (1.04, 1.5) |
| Food assistance | SNAP (past 12 mo) |  | 1.14 (1.05, 1.25) |  | 1.28 (1.10, 1.49) |
| Food security | Marginal food security | High food security | 1.28 (1.14, 1.42) |  | 1.24 (1.01, 1.52) |
|  | Low food security |  | 1.55 (1.37, 1.75) |  | 1.47 (1.21, 1.79) |
|  | Very low food security |  | 1.90 (1.65, 2.18) |  | 1.70 (1.38, 2.09) |

^1^Estimates are adjusted for all variables shown, as well as complex survey design, NHIS sampling weights, and survey year.
